# Downregulation of eEF1A/EFT3-4 Enhances Dopaminergic Neurodegeneration After 6-OHDA Exposure in *C. elegans* Model

**DOI:** 10.3389/fnins.2020.00303

**Published:** 2020-04-16

**Authors:** Pawanrat Chalorak, Permphan Dharmasaroja, Krai Meemon

**Affiliations:** Department of Anatomy, Faculty of Science, Mahidol University, Bangkok, Thailand

**Keywords:** eEF1A, Parkinson’s disease, dopaminergic neuron, *Caenorhabditis elegans*, 6-hydroxydopamine

## Abstract

Parkinson’s disease (PD) is a neurodegenerative disorder characterized by the aggregation of α-synuclein protein and selective death of dopaminergic (DA) neurons in the substantia nigra of the midbrain. Although the molecular pathogenesis of PD is not completely understood, a recent study has reported that eukaryotic translation elongation factor 1 alpha (eEF1A) declined in the PD-affected brain. Therefore, the roles of eEF1A1 and eEF1A2 in the prevention of DA neuronal cell death in PD are aimed to be investigated. Herein, by using *Caenorhabditis elegans* as a PD model, we investigated the role of *eft-3/eft-4*, the worm homolog of *eEF1A1/eEF1A2*, on 6-hydroxydopamine (6-OHDA)-induced DA neuron degeneration. Our results demonstrated that the expressions of e*ft-3* and *eft-4* were decreased in the 6-OHDA-induced worms. RNA interference (RNAi) of *eft-3* and *eft-4* resulted in dramatic exacerbation of DA neurodegeneration induced by 6-OHDA, as well as aggravated the food-sensing behavior, ethanol avoidance, and decreased lifespan when compared with only 6-OHDA-induced worms. Moreover, downregulation of *eft-3/4* in 6-OHDA-induced worms suppressed the expression of the anti-apoptotic genes, including *PI3K/age-1, PDK-1/pdk-1, mTOR/let-363*, and *AKT-1,2/akt-1,2*, promoting the expression of apoptotic genes such as *BH3/egl-1* and *Caspase-9/ced-3.* Collectively, these findings indicate that eEF1A plays an important role in the 6-OHDA-induced neurodegeneration through the phosphatidylinositol 3-kinase (PI3K)/serine/threonine protein kinase (Akt)/mammalian target of rapamycin (mTOR) pathway and that eEF1A isoforms may be a novel and effective pro-survival factor in protective DA neurons against toxin-induced neuronal death.

## Introduction

Parkinson’s disease (PD) is a progressive neurodegenerative disorder of the central nervous system (CNS) resulting from the loss of dopaminergic (DA) neurons in the substantia nigra of the midbrain, causing severe motor deficits such as tremor, rigidity, postural imbalance, and slowness of movement (Davie, 2008). Currently, there is a lack of specific and effective treatment for PD as the commonly used drug, levodopa, only palliates the symptoms and may even exacerbate the symptoms over long-term treatment. Moreover, long-term use of levodopa can cause dyskinesia in PD patients (Pandey and Srivanitchapoom, 2017). Because of the limitations of the existing PD treatment, molecular mechanisms underlying progressive DA neurons death and the discoveries of novel therapeutic agents for PD are essential for the prevention and cure of the disease.

Although molecular mechanisms of DA neurons death are not completely understood, many studies suggested that the phosphatidylinositol 3-kinase (PI3K)/serine/threonine protein kinase (Akt)/mammalian target of rapamycin kinase (mTOR) pathway is associated with the neuronal proliferation, differentiation, and programmed apoptotic cell death (Hawkins et al., 2006). Moreover, the PI3K/Akt/mTOR pathway is also associated with the neuroprotective signaling pathways in PD (Chen et al., 2013). This evidence has been supported by several studies on pharmacological treatment, such as exenatide and rotigotine, to improve PD *via* the activation of PI3K/Akt/mTOR pathway (Oster et al., 2014; Hauser et al., 2016; Athauda et al., 2019).

Eukaryotic translation elongation factor 1 alpha (eEF1A) proteins are guanosine triphosphate (GTP)-binding proteins that are transcribed in the nucleus and functions in protein synthesis. During the elongation process of protein translation, eEF1A delivers aminoacyl-tRNAs to the ribosomal A-site for binding of codon and anticodon (Dinman and Kinzy, 1997). Two isoforms, namely, the eEF1A1 and eEF1A2, can be found in mammals that are paralog and share more than 90% of human DNA (Kahns et al., 1998). Interestingly, previous studies reported that the eEF1A declined in the human brain tissues with history of PD disease progression, indicating an association of eEF1A and altered polypeptide synthesis in the corresponding area (Licker et al., 2014; Garcia-Esparcia et al., 2015). In the cellular model, upregulation of genes of *eEF1A* has been found to be associated with the increased expression of *PI3K*, *AKT*, and *mTOR* in 1-methyl-4-phenylpyridinium (MPP+)-induced cellular PD model (Khwanraj et al., 2016). Since the roles of eEF1A1 and eEF1A2 in the prevention of DA neuronal cell death in PD remain unclear, this study aims to investigate the role of eEF1A isoforms on degenerated DA neurons by using *Caenorhabditis elegans* as a PD model.

*Caenorhabditis elegans* is a eukaryotic organism with a short life cycle (Brenner, 1974), which is advantageous to monitor the progression of neurodegenerative PD diseases. *C. elegans* has been used as a PD model since it demonstrates eight well-mapped DA neurons which are subdivided into two pairs of cephalic neurons (CEP), one pair of anterior deirid neurons (ADE) in the head and the posterior deirid neurons (PDE) in the tail (Nass et al., 2002; Harrington et al., 2010). In comparison with mammals, the DA synthesis, storage, and transport mechanisms, as well as cellular apoptosis and survival PI3K/AKT/mTOR pathways, are conserved in *C. eleg*ans (Sanyal et al., 2004; Lant and Storey, 2010; Lord and Gunawardena, 2012). Moreover, *C. elegans* also share a wide homologous genome with mammals including eEF1A1; *eft-3* homolog and eEF1A2; *eft-4* homolog. Therefore, *C. elegans* is suitable to use as a model in the study of neurodegenerative diseases.

In the present study, we exposed *C. elegans* to neurotoxin, 6-hydroxydopamine (6-OHDA) to induce degeneration of DA neurons and behavioral characteristics associated with PD. This study provides evidence that the downregulation of eEF1A by RNA interference (RNAi) exhibits the morphological changes of DA neurons, deficits in dopamine-dependent behaviors, and increased lifespan in *C. elegans*. Moreover, the knockdown of eEF1A also accelerated the DA neurodegeneration induced by 6-OHDA in *C. elegans* through downregulation of the survival pathway.

## Materials and Methods

### *C. elegans* Strains, Maintenance, and Synchronization

Wild-type Bristol N2, transgenic BZ555 (*dat-1*p:GFP; green fluorescent protein expression in the DA neuronal soma and processes), SD1340 [*eft-3*p:HIS-24:mCherry; red fluorescent protein (RFP) expression in the most cells], and CU394 (*ced-3*p:GFP green fluorescent protein expression in the apoptotic cell) were obtained from the Caenorhabditis Genetics Center (University of Minnesota, United States). Strain UA202 (*dat-1*p:sid-1, *myo-2*p:mCherry; *dat-1*p:GFP) which is sensitive to RNAi specifically in DA neurons was kindly provided by Caldwell Laboratory (The University of Alabama, United States). All procedures performed in the *C. elegans* were according to the protocols approved by the Faculty of Science, Mahidol University Animal Care and Use Committee (MUSC-ACUC). All strains were cultured following standard methods (Brenner, 1974). Large populations of *C. elegans* can be grown by culturing on solid nematode growth medium (NGM) and fed with *Escherichia coli* OP50 as a food source at 20°C. Synchronized eggs were isolated from gravid worms by bleaching solution (12% NaClO and 10% 1 M NaOH), plated on NGM without bacteria and then incubated at 20°C overnight to obtain newly hatched animals or L1 larvae. To acquire L3 larvae, synchronized L1 larvae were transferred onto NGM plates containing *E. coli* OP50 and incubated for 20–24 h at 20°C.

### RNAi Treatment

Bacterial RNAi feeding constructs of *eft-3* and *eft-4* were obtained from the Vidal laboratory library (Source BioScience, Nottingham, United Kingdom). They were isolated and grown overnight in Luria broth medium plus 100 μg/ml ampicillin (Sigma Aldrich, St. Louis, MO, United States) at 37°C. The cultured bacteria were seeded onto NGM plates containing 1 mM isopropyl β-D-1-thiogalactopyranoside (IPTG; Sigma Aldrich, St. Louis, MO, United States) to induce dsRNA expression, and then, these plates were allowed to dry overnight at room temperature and stored at 4°C.

### *C. elegans* Dopaminergic Neurodegeneration Assay

The *C. elegans* were induced by the neurotoxin 6-OHDA (Sigma Aldrich, St. Louis, MO, United States) to selectively degenerate the DA neurons. Synchronized L3 larvae were treated in a solution containing diluted OP50 mixed with 10, 25, and 50 mM 6-OHDA and 2, 5, and 10 mM ascorbic acid, respectively. The solution was mixed gently with pipette every 10 min for 1 h at 20°C. After incubation, the worms were washed by M9 buffer for three to four times. Induced worms were then retransferred onto RNAi bacterial plates and 0.04 mg/ml 5-fluoro-2′-deoxyuridine (FUDR, Sigma, St. Louis, MO, United States) for inhibiting progeny development and treated for 72 h before performing in subsequent assays. L4440 [empty vector (EV)] bacteria NGM plates were used as a control. The experiment was performed independently for three times (*n* = 40–50 animals/group per replicate).

### Imaging and Quantification Analysis

After treating the worms with various conditions of 6-OHDA, the adult hermaphrodites were washed three times with M9 buffer to remove the bacteria from the nematode cuticle. They were loaded onto 2% agarose-padded slides. Worms were anesthetized by 2% sodium azide and sealed with coverslips. The immobilized worms were observed and photographed under a fluorescence microscope (BX53, Olympus Corp., Tokyo, Japan). Fluorescence intensity was measured in 40–50 randomly selected worms by ImageJ software [National Institutes of Health (NIH), Maryland, United States]. For phenotype analysis of DA neurons, the observer was blinded to the genotype and the treatment of the worms.

### Food-Sensing Behavior

This assay was performed to test the food-sensing function controlled by DA neurons. Normally, the worms move slowly in bacterial lawn when compared with worms in plates without bacteria (Sawin et al., 2000). In brief, *E. coli* OP50 were spread on the center of NGM plates, and the bacteria-containing plates were incubated at 37°C overnight. Wild-type L3 larvae nematodes were firstly treated with either 6-OHDA with empty vector bacteria or 6-OHDA with RNAi bacteria for 72 h at 20°C and then washed three times with M9 buffer. Worms were transferred to the plates with or without bacterial supply and settled for 5 min. The worm body bending was recorded and counted in 30-s intervals. The body bending in the plates with and without bacteria was counted and compared in each group. Percentage of basal slowing rate was calculated as the difference in the rate of movement on bacterial lawn versus outside bacterial lawn divided by the rate of movement outside bacterial lawn (*n* ≥ 30 animals/group per replicate).

### Ethanol Avoidance Assay

Adult treated worms were washed three times with M9 buffer and transferred to the center of NGM assay plates. Assay plates were divided into four quadrants; two quadrants seeded with 50 μl ethanol and two quadrants seeded with 50 μl distilled water. After 30 min, the numbers of worms were scored in each quadrant. Ethanol avoidance index was calculated as the difference in number of worms in control quadrants versus in ethanol quadrants divided by the total number of worms (Cooper et al., 2015; Maulik et al., 2017) (*n* ≥ 30 worms/group per replicate).

### Lifespan Assay

After being treated with RNAi or EV bacteria, synchronized L3 stage N2 were induced with 6-OHDA for 1 h and transferred to RNAi plates containing various conditions, EV bacteria, and 0.04 mg/ml FUdR plates at 20°C. The numbers of live and dead worms were counted and recorded daily until all worms died, then the mean lifespan and percent survival were calculated. Dead worms were determined when they did not respond to touch by a platinum wire and showed no pharyngeal pumping. Censored worms were the worms with internally hatched progeny or extruded gonad which were excluded from the experiment. Three independent replicates were conducted for each treatment with approximately 30–40 animals per replicate, so more than 100 animals were analyzed.

### Quantitative RT-PCR

Total RNA was synthesized using the RNA extraction kit (Qiagen, Germany) following the manufacturer’s protocol. The RNA samples were stored at −80°C until use. For quantitative gene expression analyses, high-capacity complementary DNA (cDNA) was generated from 2 μg of RNA using the iScriptTMReverse Transcription Supermix for RT-qPCR (Bio-Rad, Foster City, CA, United States). Then, cDNA was diluted to a ratio of 1:10 with SsoFast^*TM*^ EvaGreen^®^ Supermix with Low ROX qRT-PCR (Bio-Rad, Foster City, CA, United States), mixed with forward and reverse primers of specific genes (Table 1). Real-time PCR was first performed by holding the sample at 95°C for 30 s. Then, the PCR sample was set for denaturing at 95°C for 5 s and to annealing/extension at 60°C for 30 s. After 44 cycles repeat, the sample was then heated up to 95°C for melt curve analysis. Eventually, EvaGreen fluorescence was detected by the CFX96 Touch Real-time PCR detection system (Bio-Rad, Foster City, CA, United States), and Cq values were obtained. All targeted genes were measured in triplicate, and three independent biological triplicates were detected in each condition. The Cq values were then calculated *via* 2-(ΔΔCq) equation representing relative fold change in the expression of each gene. Relative mRNA expression levels were normalized using reference internal control gene, *act-1.*

**TABLE 1:** Primer lists used in this study.

Forward (5′→3′)		Reverse (5′→3′)
**EFT isoforms**		
*eft-2*	TTTACTCTTGTCACGCCGCT	TCCATAAGCGCACGGATCTC
*eft-3*	GGACGTGTTGAGACCGGAAT	TGACGGAGACGTTCTTGACG
*eft-4*	CATGGTTCAAGGGA TGGGCT	ATTCCGGTCTCAACACGTCC
**Apoptosis mediators**		
*egl-1*	CTAGCAGCAATGTGCGATGAC	GGAA GCATGGGCCGAGTAG
*ced-9*	TGCTCAGGACTTGCCATCAC	TTGACTCTCCGATGGACATTCTT
*ced-4*	AAGTCGAGGATTAGTCGGTGTTG	AGAGCCATTGCGAGTGACTTG
*ced-3*	TCAACGCGGCAAATGCT	GCCTGCACAAAAACGATTTTC
**Survival mediators**		
*daf-2*	GGCCGATGGACGTTATTTTG	TTCCACAGTGAAGAAGCCTGG
*age-1*	TCTGATTGCTGGACACGGAC	CGATGGGCGTTCTCGCATT
*let-363*	GCCACTCTCTGATTACCCTGT	GTGAGCCGCGTGTTTCAAAT
*pdk-1*	AGTCCTGCACGCCTACATTC	CGTTAGACGGTGTTGATGGC
*akt-1*	TCACCGATGCGATATTGTCT	AACTCCCCACCAATCAACAC
*akt-2*	ATCAGCCGTTACCAGAGC	AAGGTTCCTTGACCGAGA
**Housekeeping gene**		
*act-1*	AGGTTGCCGCTCTTGTTGTA	CGTGGTCTTCCGACAATGGA

### Statistical Analysis

All assays were completed with a minimum of three biological replicates. All data were presented as mean ± standard error of the mean (SEM). Differences among groups were determined by one-way analysis of variance (ANOVA) following the Tukey–Kramer test for multiple comparisons. For grouped analyses, a two-way ANOVA series was used with Tukey’s multiple comparison for *post hoc* test. Survival plots were compared using the log-rank test. Probability levels (*p*-value) of <0.05 were considered statistically significant. All statistical analyses were determined by GraphPad Prism software 7 (GraphPad Software Inc.).

## Results

### Dopaminergic Neurons Loss and Reduction of *eft-3* and *eft-4* Induced by 6-Hydroxydopamine Exposure

**FIGURE 1:**
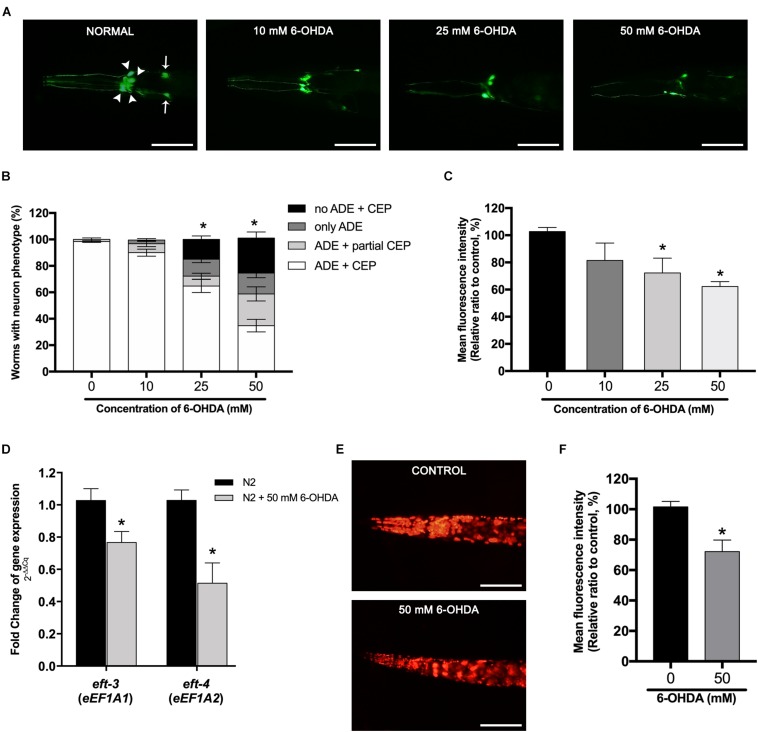
Effects of 6-hydroxydopamine (6-OHDA) exposure on dopaminergic (DA) neurons and the mRNA expression of the *eft-3* and *eft-4* in *Caenorhabditis elegans.* **(A)** Green fluorescent protein (GFP) expression pattern in DA neurons of control transgenic BZ555 strain, and 10, 25, and 50 mM 6-OHDA-treated worms. In normal BZ555 worms, white arrowheads point to cell bodies of four cephalic neurons (CEP), while white arrows indicate the location of two anterior deirid neuron (ADE) cell bodies. Scale bar, 50 μm. **(B)** Graphical representation for percentage of DA neurons phenotype worms. Animals possessing all neurons were scored as “ADE + CEP” (white bar), those with partial loss of CEP but intact ADE neurons as “ADE + partial CEP” (light gray bar), those with complete loss of CEP but intact ADE neurons as “only ADE” (dark gray bar), and those with complete loss of DA head neurons as “no ADE + CEP” (black bar). The asterisk (*) indicates the significant difference of percent worm with normal ADE + CEP phenotype between untreated and 6-OHDA-treated worms (*p* < 0.05). **(C)** Graphical representation for mean fluorescence intensity (MFI) of GFP expression in DA neurons of BZ555 strain as measured by using ImageJ software. **(D)** Fold change of mRNA expression levels of *eft-3* and *eft-4* between normal N2 worms and 50 mM 6-OHDA-treated worms. **(E,F)** GFP expression and graphical representation for MFI of red fluorescent protein (RFP)-tagged *eft-3* in SD1340 strain and 50 mM 6-OHDA-treated worms. The data represent the mean ± SEM of three independent biological replicates, each with 40–50 animals. For **(C–F)**, the asterisk (*) indicates a significant difference between the untreated and 6-OHDA-treated worms (*p* < 0.05).

In transgenic BZ555 strain worms, morphological patterns of DA neurons were fluorescently labeled by the expression of the dopamine transporter marker P*dat-1*:GFP (Figure 1A, control). Selective degeneration of *C. elegans* DA neurons could be accomplished by the exposure of 6-OHDA. Results showed that the exposure to 10, 25, and 50 mM of 6-OHDA gradually damaged cell bodies in CEPs and ADEs and fragmented neuronal processes of anterior CEP in a dose-dependent manner (Figure 1A). We defined the DA neurodegeneration by determining GFP expression as the following indexes: (1) ADE + CEP, (2) ADE + partial CEP, (3) only ADE, and (4) no ADE + CEP. We found that the percent of worms possessing all ADE and CEP significantly reduced to 64.8% ± 4.97% and 34.8% ± 4.75% when exposed to 25 and 50 mM 6-OHDA, respectively (Figure 1B). Moreover, the DA neuron viability in BZ555 was also evaluated by analyzing the mean fluorescence intensity (MFI) of GFP expression and the morphology of DA neurons. Our results showed that the percent relative MFI of 25 and 50 mM 6-OHDA-treated group significantly reduced to 73.96% ± 7.51% and 62.23% ± 2.12%, respectively (Figure 1C). However, in 10 mM 6-OHDA exposure, there were non-significant changes in both GFP fluorescence and apparent dopamine–neuronal morphological changes of ADE and CEP compared to the control group.

To further determine the effect of 6-OHDA exposure on the expression of eEF1A isoforms in the *C. elegans* PD model, we investigated the mRNA expression changes of *eft-3* and *eft-4* (homolog of mammalian *EEF1A1* and *EEF1A2*, respectively) after exposure to 50 mM 6-OHDA. Quantitative RT-PCR showed that *eft-3* and *eft-4* mRNA expression levels were significantly reduced to 0.76 ± 0.07 fold and 0.51 ± 0.12 fold in 6-OHDA-treated worms when compared with normal worms (Figure 1D). Moreover, using transgenic strain SD1340 for the expression of fusion *eft-3*:RFP, we found obvious downregulation of RFP expression in the 6-OHDA-treated group (Figure 1E), with MFI significantly reduced to approximately 72.17% ± 4.34% (*P* < 0.05) when compared to the untreated worms (Figure 1F).

### RNAi Knockdown of *eft-3* and *eft-4* Accelerated Dopamine Neurodegeneration Caused by 6-Hydroxydopamine

**FIGURE 2:**
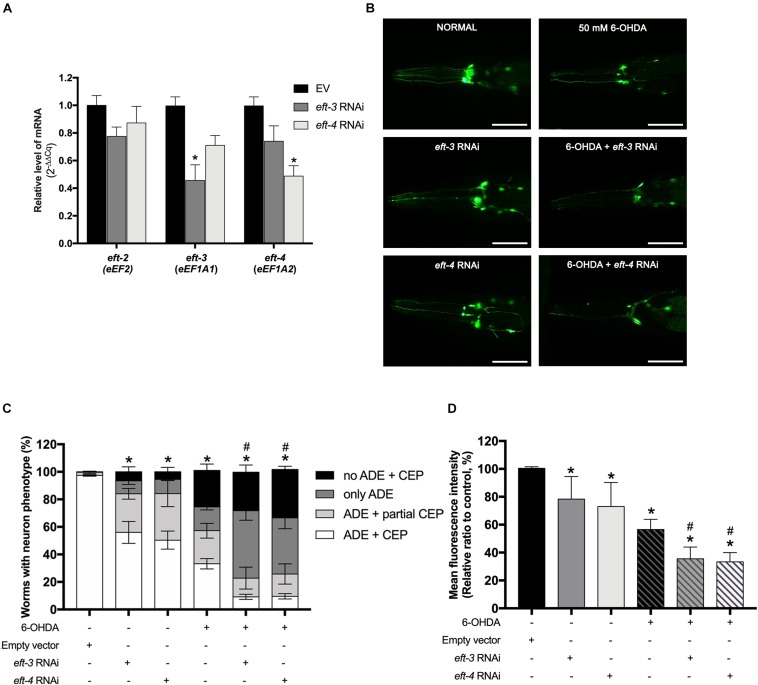
Effects of *eft-3*/*eft-4* RNA interference (RNAi) and/or 6-hydroxydopamine (6-OHDA) exposure on the dopaminergic (DA) neurons in *Caenorhabditis elegans.* **(A)** Fold change of mRNA expression levels of *eft-3* and *eft-4* between empty vector (EV) RNAi and *eft-3* or *eft-4* RNAi-treated worms to ensure specific knockdown. The asterisk (*) indicates a significant difference between the EV RNAi and *eft-3* or *eft-4* RNAi-treated worms (*p* < 0.05). **(B)** Green fluorescent protein (GFP) expression pattern in DA neurons of normal UA202 strain, *eft-3*/*eft-4* RNAi-treated, 6-OHDA-treated, and co-treatment between *eft-3/eft-4* RNAi and 6-OHDA-treated worms. Scale bar, 50 μm. **(C)** Graphical representation for percentage of DA neurons phenotype worms. The asterisk (*) indicates a significant difference of percent worm with normal ADE + CEP phenotype between EV RNAi-treated and *eft-3/eft-4* RNAi and/or 6-OHDA-treated worms (*p* < 0.05). The hash (#) indicates a significant difference of percent worms carrying normal ADE + CEP phenotype between only 6-OHDA-treated and combined 6-OHDA and RNAi-treated worms (*p* < 0.05). **(D)** Graphical representation for mean fluorescence intensity (MFI) of GFP expression in DA neurons as measured by using ImageJ software. The asterisk (*) indicates a significant difference between the EV RNAi-treated and *eft-3/eft-4* RNAi and/or 6-OHDA-treated worms (*p* < 0.05). The hash (#) indicates the significant difference between only 6-OHDA-treated and combined 6-OHDA with *eft-3* or *eft-4* RNAi-treated worms (*p* < 0.05).

*Caenorhabditis elegans eft-3 or eft-4* homozygous null mutant generated an abnormal development involving embryonic viability, fertility, and germline maintenance (Gönczy et al., 2000; Maeda et al., 2001). Therefore, to determine the function of *eft-3 and eft-4* on DA neurons without interfering with the normal cellular process, we performed the RNAi knockdown in *C. elegans* UA202 strain to assay for DA neurodegeneration. After RNAi knockdown, the levels of *eft-3* or *eft-4* mRNA expression were significantly reduced, while *eft-2* mRNA level did not change, indicating successful knockdown experiments in these treated worms (Figure 2A). Decreasing of *eft-3* or *eft-4* expression generated fragmented DA neuronal processes, blebbing neurites, and shrinkage of cell bodies (Figure 2B). Quantification analysis of GFP expression on DA neurons revealed that knocking down *eft-3* and *eft-4* caused a significant decrease of the percentage of worms carrying normal DA neurons at 56.00% ± 7.97% and 50.40% ± 6.54%, respectively (Figure 2C). Meanwhile, the knockdown of *eft-3* and *eft-4* also resulted in a significant decrease of the MFI of DA neurons at 78.36% ± 7.26% and 73.04% ± 7.68%, respectively, when compared with EV control (Figure 2D).

To determine the roles of *eft-3* and *eft-4* in DA neurodegeneration in PD, we further examined the effects of *eft-3* or *eft-4* RNAi on 6-OHDA vulnerability in *C. elegans*. As expected, worms treated with 50 mM 6-OHDA and EV exhibited DA neurons loss (Figure 2B). Moreover, co-treatment of *eft-3* or *eft-4* RNAi with 6-OHDA worsened DA neuronal losses (Figure 2B). By quantifying the morphology of DA neurons, the results showed that 6-OHDA co-treatment with *eft-3* or *eft-4* RNAi caused greatly significant decreases of the percentage of worms carrying normal DA neurons at 9.20% ± 1.90% and 9.60% ± 2.92%, respectively (Figure 2C). The MFI of DA neurons in 6-OHDA co-treatment with *eft-3* or *eft-4* RNAi condition also significantly decreased in the mean intensity at 35.51% ± 3.80% and 33.31% ± 2.98%, respectively, when compared with 6-OHDA treatment alone (Figure 2D).

### Decreasing of *eft-3* or *eft-4* Caused Deficits in Food Sensing and Ethanol Avoidance Behaviors

**FIGURE 3:**
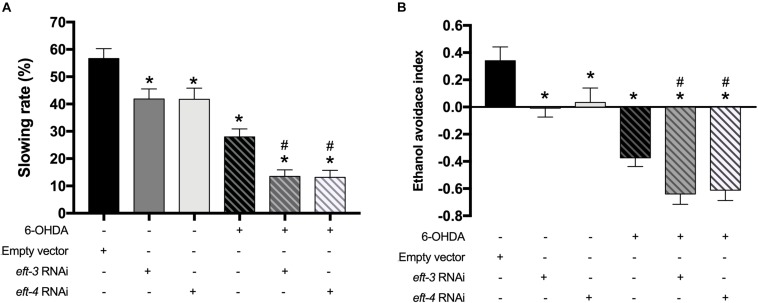
Effects of *eft-3*/*eft-4* RNA interference (RNAi) and/or 6-hydroxydopamine (6-OHDA) exposure on dopamine-dependent behaviors. **(A)** Graphical representation of slowing rate of body bending and **(B)** ethanol avoidance index in the UA202 strain. The asterisk (*) indicates a significant difference between the empty vector (EV) RNAi-treated and *eft-3/eft-4* RNAi and/or 6-OHDA-treated worms (*p* < 0.05). The hash (#) indicates a significant difference between only 6-OHDA-treated and the combined 6-OHDA with *eft-3* or *eft-4* RNAi-treated worms (*p* < 0.05).

We further examined the effects of *eft-3* and *eft-4* RNAi on the dopamine-dependent behaviors: food-sensing behavior or basal slowing rate (Sawin et al., 2000) and ethanol avoidance (Cooper et al., 2015) in *C. elegans* which are controlled by DA neurons. Normally, when worms come across the bacteria lawn or food source, they reduce body bending frequency to feed themselves. UA202 worms revealed 56.66% ± 3.63% reduction in bending frequency when in contact with bacteria lawn. 6-OHDA-treated worms, however, failed to reduce the bending frequency due to the loss of the food-sensing system. For the UA202 treated with 6-OHDA, the basal slowing rate was significantly reduced to 28.79% ± 2.78% when compared with the untreated N2 group (Figure 3A). The single *eft-3* or *eft-4* RNAi-treated worms also displayed a significant reduction in slowing response to 42.88% ± 3.51% and 41.63% ± 3.98%, respectively. Moreover, the combination of 6-OHDA and *eft-3* or *eft-4* RNAi treatment significantly reduced the basal slowing behavior to 13.39% ± 2.29% and 13.61% ± 2.35%, respectively, compared with normal and 6-OHDA treatment alone (Figure 3A). Similarly, *eft-3* or *eft-4* RNAi-treated worms also exhibited the significant ethanol avoidance deficit by decreasing the ethanol avoidance index to -0.01 and 0.03 when compared to normal worms. The combined *eft-3* or *eft-4* RNAi and 6-OHDA exposure also caused more significant decrease to −0.64 and −0.61 compared with both EV control and only 6-OHDA exposure (Figure 3B).

### Decreasing of *eft-3* or *eft-4* Enhanced 6-Hydroxydopamine-Induced Shortening of Lifespan

**FIGURE 4:**
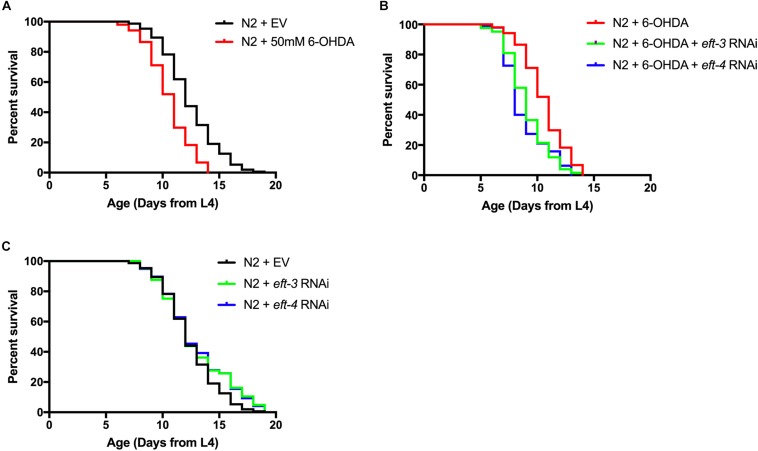
Effects of *eft-3*/*eft-4* RNA interference (RNAi) and/or 6-hydroxydopamine (6-OHDA) exposure on lifespan in N2 *Caenorhabditis elegans.* Cumulative survival plots of 6-OHDA-treated **(A)**, co-treatment of *eft-3/eft-4* RNAi and 6-OHDA-treated worms **(B)**, and empty vector (EV) RNAi-treated and *eft-3*/*eft-4* RNAi-treated worms **(C)**. Quantitative data representing mean lifespan, maximum lifespan, percentage of increased lifespan, and significance are shown in Table 2.

We further examined the effects of *eft-3* or *eft-4* RNAi and co-treatment with 6-OHDA on the lifespan of *C. elegans.* The mean lifespan of N2 worms was approximately 12.54 ± 0.20 days. In 6-OHDA-induced worms, the mean lifespan was significantly reduced to 10.74 ± 0.18 days and shortened by 14.35% from normal worms (Figure 4A). Besides, knocking down *eft-3* or *eft-4* combined with 6-OHDA treatment caused a further shortening lifespan with 12.98 and 12.20% reduction compared with 6-OHDA treatment only (Figure 4B and Table 2). On the other hand, the *eft-3* or *eft-4* RNAi-treated worms showed a non-significant increase of mean lifespan at 3.76%, 4.84% compared with normal worms, respectively (Figure 4C and Table 2).

**TABLE 2:** Mean lifespan, maximum lifespan, percentage of increase lifespan and significance *P*-values.

Treatment	Mean lifespan (day)	Maximum lifespan	% increase lifespan	Significant (*P*-value)
N2 + EV	12.54 ± 0.20	19	–	–
N2 + *eft-3* RNAi	13.01 ± 0.29	19	3.76 (compared with N2)	*P < 0.05
N2 + *eft-4* RNAi	13.14 ± 0.30	19	4.84 (compared with N2)	*P < 0.05
N2 + 6-OHDA	10.74 ± 0.18	14	−14.35 (compared with N2)	***P < 0.001
N2 + 6-OHDA + *eft-3* RNAi	9.35 ± 0.17	15	−12.98 (compared with N2 + 6-OHDA)	***P < 0.001
N2 + 6-OHDA + *eft-4* RNAi	9.43 ± 0.22	16	−12.20 (compared with N2 + 6-OHDA)	***P < 0.001

### Decreasing of *eft-3* or *eft-4* Affected the Apoptotic Gene Expression

**FIGURE 5:**
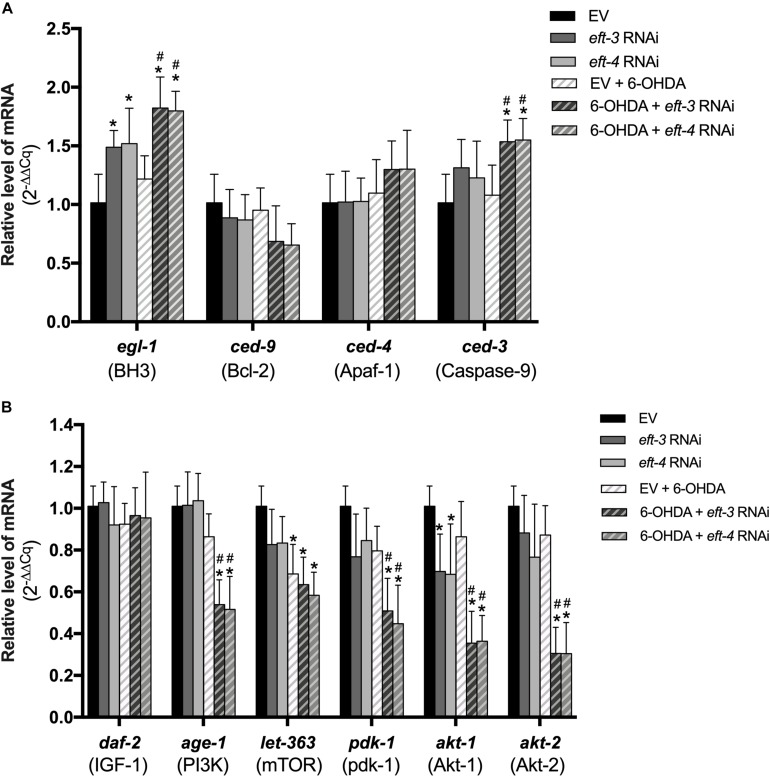
Effects of *eft-3*/*eft-4* RNA interference (RNAi) and/or 6-hydroxydopamine (6-OHDA) exposure on apoptosis and survival modulation. **(A)** Fold change of mRNA expression levels of apoptosis signaling genes including *egl-1*, *ced-3*, *ced-4*, and *ced-9* in empty vector (EV) RNAi-treated, *eft-3*/*eft-4* RNAi-treated, 6-OHDA-treated, and co-treatment of *eft-3/eft-4* RNAi and 6-OHDA-treated groups. **(B)** Fold change of mRNA expression levels of survival signaling genes including *daf-2*, *age-1*, *let-363*, *pdk-1*, *akt-1*, and *akt-2* in EV RNAi-treated, *eft-3*/*eft-4* RNAi-treated, 6-OHDA-treated, and co-treatment of *eft-3/eft-4* RNAi and 6-OHDA-treated groups. All data represent the mean ± SEM of three independent biological replicates. The asterisk (*) indicates a significant difference between the EV RNAi-treated and *eft-3/eft-4* RNAi and/or 6-OHDA-treated worms (*p* < 0.05). The hash (#) indicates a significant difference between only 6-OHDA-treated and combined 6-OHDA with *eft-3* or *eft-4* RNAi-treated worms (*p* < 0.05).

Previously, apoptotic cell death has been linked with DA neuronal loss and the expression of eEF1A (Li et al., 2010; Zeng et al., 2018), hence we sought to determine whether the underlying mechanisms of DA neuron loss is mediated by apoptosis signaling pathway. mRNA transcript levels of genes involved with apoptosis were determined in normal, *eft-3* or *eft-4* RNAi-exposed, 6-OHDA-induced, and combined-treated worms. We used qPCR to analyze the mRNA levels of *egl-1*, *ced-3*, *ced-4*, and *ced-9*, which are associated with apoptosis in *C. elegans.* We found that the expression levels of *egl-1*, *ced-3*, *ced-4*, and *ced-9* were not significantly changed in 6-OHDA-treated worms compared to those in untreated worms (Figure 5A). The level of *egl-1* mRNA was upregulated following the knockdown of *eft-3* or *eft-4* with 1.48 ± 0.14 fold and 1.52 ± 0.30 fold compared with control. In co-treatment of *eft-3* or *eft-4* RNAi and 6-OHDA-treated worms, the expression of *egl-1* showed more significant increase when compared with both EV worms and only 6-OHDA-treated worms. Moreover, the expression level of *ced-3* was slightly increased in *eft-3* or *eft-4* RNAi-treated worms with 1.31 ± 0.24 fold and 1.42 ± 0.31 fold, respectively. The combined *eft-3* or *eft-4* RNAi and 6-OHDA treatment caused a significant increase of *ced-3* mRNA level compared with both EV control and only 6-OHDA exposure (Figure 5A).

To confirm the apoptosis process in the worms, we further analyzed the CED-3 expression using transgenic CU394 strain (P_*ced–*__3_:ced-3:gfp). We found that the *eft-3/eft-4* RNAi- treated worms did not show the change in the MFI of CED-3 expression in *C. elegans* when compared with the EV RNAi (Supplementary Figures S1A,B). However, the MFI of CED-3 was upregulated in combined *eft-3* or *eft-4* RNAi and 6-OHDA-treated worms compared with both EV control and 6-OHDA treatment alone (Supplementary Figures S1A,B).

### Decreasing of *eft-3* or *eft-4* Affected the Survival Gene Expression

Having revealed a role for eEF1A in PI3K/AKT/mTOR mechanism in several cellular models (Amiri et al., 2006; Khwanraj et al., 2016), we sought to examine whether the *eft-3* and *eft-4* mediate neuroprotection through the cell survival signaling, PI3K/AKT/mTOR, in 6-OHDA-induced *C. elegans* PD model. We performed real-time qPCR to analyze the mRNA levels of *daf-2*, *age-1*, *let-363*, *pdk-1*, *akt-1*, and *akt-2*, which are homologs with cell-survival signaling genes in mammals. Results showed that knockdown of *eft-3* or *eft-4* significantly lowered only the *akt-1* mRNA expression compared with control. However, the mRNA expression of only *let-363* was significantly reduced in the 6-OHDA-treated group compared with normal. The co-treatment condition of 6-OHDA and *eft-3* or *eft-4* knockdown caused significant decreases in mRNA expression of *age-1*, *let-363*, *pdk-1*, *akt-1*, and *akt-2* compared with both EV control and 6-OHDA treatment alone (Figure 5B).

## Discussion

In the present study, we reported for the first time the roles of *eft-3/4* (homolog of mammalian eEF1A1/2) in *C. elegans* model of PD. Our data demonstrated that the nematodes exposed to 50 mM 6-OHDA display PD-like phenotype together with the decreased expression of EFTs. Downregulation of *eft-3* or *eft-4* enhanced 6-OHDA-induced DA neurodegeneration, aggravated food-sensing and ethanol avoidance behaviors, and shortened lifespan. Moreover, the molecular signaling analysis showed that the combination of EFT downregulation and 6-OHDA exposure could increase the expression of *egl-1* and *ced-3* in the apoptosis pathway and also greatly decrease the expression of genes in the PI3K/AKT/mTOR survival pathway, *age-1*, *let-363*, *pdk-1*, *akt-1*, and *akt-2*.

Parkinson’s disease is the neurodegenerative disease associated with the selective loss of DA neurons in the substantia nigra (Hartmann, 2004). It has been proposed that the major loss of DA neurons may come from chronic exposure to neurotoxins (Bové and Perier, 2012). In the present study, we showed that the exposure to 6-OHDA induced the PD-like phenotypes in a dose-dependent manner in *C. elegans*. 6-OHDA caused degeneration in DA neurons which led to abnormality in the physiological function correlated with the degeneration of the DA neuron in *C. elegans*, including food-sensing behavior and the shortened lifespan. These results are consistent with the previous studies that have reported 6-OHDA-induced parkinsonism in both *in vitro* and *in vivo* models (Latchoumycandane et al., 2011; Bagga et al., 2015; Chalorak et al., 2018). 6-OHDA selectively induced the toxicity in the DA neurons by entering the cell through dopamine transporter which expressed exclusively in the DA neuronal cell surface (Nass et al., 2002). Once entering into the neurons, 6-OHDA increased oxidative stress and triggered the signaling pathways that eventually led to cell death (Offenburger et al., 2018). Furthermore, in this study, we found that 6-OHDA also decreased the expression of *eft-3* and *eft-4* in both protein and RNA expression levels. Similarly, decreasing of eEF1A expression has been found in PD brain patients (Garcia-Esparcia et al., 2015). Alteration of protein translation has been proposed to be a contributing factor to PD pathogenesis (Moreno et al., 2012; Taymans et al., 2015; Zhou et al., 2019). Thus, our results encourage the possibility that downregulation of protein translation regulators, eEF1As, is associated with PD and toxin-induced PD model.

eEF1A1 and eEF1A2 proteins are GTP binding proteins that deliver the amino acids during protein translation (Zhu et al., 2001). The downregulation of eEF1As has been previously found in the substantia nigra and frontal cortex of the PD patients, although the involvement of eEF1A and PD incidence remains unclear (Licker et al., 2014; Garcia-Esparcia et al., 2015). To understand the role of eEF1A in PD pathophysiology, we investigated the effects of *eft-3* or *eft-4* knockdown on *C. elegans* PD model. In this study, we found that the downregulation of *eft-3* and *eft-4* caused the degenerative and misplaced DA neurons in *C. elegans*. Knocking down these genes in *C. elegans* also exhibited the dysfunction of the DA neurons as seen in the food-sensing behavior assay. Our results are consistent with the previous reports that eEF1A1/2 is necessary for neurite outgrowth and neuronal survival (Hashimoto and Ishima, 2011; Sun-Jung et al., 2012). Moreover, knocking down *eft3/4* in 6-OHDA-exposed nematodes worsened the DA neuronal degeneration caused by 6-OHDA exposure alone and greatly aggravated the dopamine-dependent behaviors. These results suggested that *eft3/4* may play an important role to sustain DA neurons and their function against neurotoxin.

Although knocking down either *eft-3* or *eft-4* has similar effects to 6-OHDA-induced DA degeneration, the lifespan of *eft-3* or *eft-4* RNAi-treated worms was slightly increased compared with the normal worms. However, combined knocking down *eft3/4* and 6-OHDA exposure could shorten the lifespan compared with 6-OHDA-induced alone. Previous studies on the interaction between genetic and environmental exposure has shown that many gene mutations could increase the sensitivity to the neurotoxins and cause more severe defects in animal models (Meulener et al., 2005; Ray et al., 2014; Zeng et al., 2018). From these data, it might be possible that the *eft3/4* knockdown enhanced the 6-OHDA-induced DA neurodegenerations in *C. elegans*.

There are two signaling pathways associated with 6-OHDA-induced DA neuron degeneration in *C. elegans*: (i) the apoptotic signaling molecules and (ii) the anti-apoptotic molecules (Nass et al., 2002). Previously, the microarray analysis of DA neurons from PD patients showed that several signaling pathways are dysregulated. Among these pathways, PI3K/Akt/mTOR signaling pathway was found to be associated with all dysregulation (Elstner et al., 2011). Here, we revealed that 6-OHDA downregulates the mTOR-like *let-363* gene expression without alteration of BH3-like *egl-1*, Apaf-1-like *ced-4*, and caspase-9-like *ced-3* expressions. This observation is similar to previous reports indicating that the 6-OHDA-induced DA cell death is associated with the downregulation of mTOR (Zhang et al., 2017). Moreover, previous studies suggested that 6-OHDA induced DA neuron death independently from apoptosis signaling, *ced-3* and *ced-4*, in *C. elegans* (Nass et al., 2002). Our study showed that the knockdown of *eft3/4* suppressed the *akt-1* but not *akt-2* expression and promoted the gene expression of *egl-1* and *ced-3* which are the key apoptotic signaling proteins (Amiri et al., 2006). Previously, eEF1A2 has been shown to possess the anti-apoptosis effect in mouse plasmacytoma cell lines *via* activation of PI3K/Akt (Li et al., 2010). Although the effects on gene expression of 6-OHDA and *eft-3/4* gene silencing were different, the anti-apoptotic genes were strongly suppressed when both treatments were combined. The previous findings showed that the downregulation of eEF1A1 causes the neurons to be more vulnerable to the external insults (Vera et al., 2014; Davies et al., 2017), and our results further showed that the downregulation of eEF1A2 also increases sensitivity to external insults. Our data encourage the fact that the downregulation of *eft-3/4* attenuates the neurons to be more vulnerable to neurotoxin by suppression of the anti-apoptotic genes as well as the promotion of some apoptotic gene expressions.

## Conclusion

Our study reported that the downregulation of *eft3* or *eft-4* by RNAi impairs the survival of DA neurons and food-sensing and ethanol avoidance behaviors in *C. elegans*. The downregulation promoted a pro-apoptotic gene *egl-1* expression and decreased expression of pro-survival gene *akt-1*. Moreover, *eft3/4*-knocked down worms displayed more severely in DA neuronal system when being exposed in combination with 6-OHDA. Gene expression analysis showed that the combination of *eft3/4* downregulation and 6-OHDA decreases several pro-survival gene expressions including *age-1*, *akt1/2*, and *let-36*3. These results suggested that the downregulation of *eft3/4* may facilitate the devastating effects of the neurotoxin 6-OHDA, and this can be a contributing factor of toxin-induced PD in humans.

## Data Availability Statement

The datasets generated for this study are available on request to the corresponding author.

## Ethics Statement

All animal procedures used in the present study were performed in the *C. elegans* model which were approved by the Faculty of Science, Mahidol University Animal Care and Use Committee (MUSC-ACUC).

## Author Contributions

All authors listed contributed immensely to this study. PC performed the experiments, conducted the data analysis, and wrote the manuscript. KM and PD interpreted the data, suggested and put forward the idea, and reviewed and edited the manuscript. All authors read and approved the final version of the manuscript.

## Conflict of Interest

The authors declare that the research was conducted in the absence of any commercial or financial relationships that could be construed as a potential conflict of interest.

## References

[B1] Amiri A. Noei F. Jeganathan S. Kulkarni G. Pinke D. E. Lee J. M. (2006). eEF1A2 activates Akt and stimulates Akt-dependent actin remodeling, invasion and migration. *Oncogene* 26:3027. 10.1038/sj.onc.1210101 17130842

[B2] Athauda D. Gulyani S. Karnati H. K. Li Y. Tweedie D. Mustapic M. (2019). Utility of neuronal-derived exosomes to examine molecular mechanisms that affect motor function in patients with parkinson disease: a secondary analysis of the exenatide-PD trial. *JAMA Neurol.* 76 420–429. 10.1001/jamaneurol.2018.4304 30640362 PMC6459135

[B3] Bagga V. Dunnett S. B. Fricker R. A. (2015). The 6-OHDA mouse model of Parkinson’s disease – Terminal striatal lesions provide a superior measure of neuronal loss and replacement than median forebrain bundle lesions. *Behav. Brain Res.* 288 107–117. 10.1016/j.bbr.2015.03.058 25841616

[B4] Bové J. Perier C. (2012). Neurotoxin-based models of Parkinson’s disease. *Neuroscience* 211 51–76. 10.1016/j.neuroscience.2011.10.057 22108613

[B5] Brenner S. (1974). The genetics of *Caenorhabditis elegans*. *Genetics* 77 71–94. 4366476 10.1093/genetics/77.1.71 PMC1213120

[B6] Chalorak P. Jattujan P. Nobsathian S. Poomtong T. Sobhon P. Meemon K. (2018). Holothuria scabra extracts exhibit anti-Parkinson potential in *C. elegans*: a model for anti-Parkinson testing. *Nutr. Neurosci.* 21 427–438. 10.1080/1028415X.2017.1299437 28276260

[B7] Chen A. Xiong L. J. Tong Y. Mao M. (2013). Neuroprotective effect of brain-derived neurotrophic factor mediated by autophagy through the PI3K/Akt/mTOR pathway. *Mol. Med. Rep.* 8 1011–1016. 10.3892/mmr.2013.1628 23942837

[B8] Cooper J. F. Dues D. J. Spielbauer K. K. Machiela E. Senchuk M. M. Van Raamsdonk J. M. (2015). Delaying aging is neuroprotective in Parkinson’s disease: a genetic analysis in *C. elegans* models. *NPJ Parkinsons Dis.* 1 15022–15022. 10.1038/npjparkd.2015.22 28725688 PMC5516561

[B9] Davie C. A. (2008). A review of Parkinson’s disease. *Br. Med. Bull.* 86 109–127. 10.1093/bmb/ldn013 18398010

[B10] Davies F. C. J. Hope J. E. McLachlan F. Nunez F. Doig J. Bengani H. (2017). Biallelic mutations in the gene encoding eEF1A2 cause seizures and sudden death in F0 mice. *Sci. Rep.* 7 46019–46019. 10.1038/srep46019 28378778 PMC5380952

[B11] Dinman J. D. Kinzy T. G. (1997). Translational misreading: mutations in translation elongation factor 1alpha differentially affect programmed ribosomal frameshifting and drug sensitivity. *RNA* 3 870–881. 9257646 PMC1369532

[B12] Elstner M. Morris C. M. Heim K. Bender A. Mehta D. Jaros E. (2011). Expression analysis of dopaminergic neurons in Parkinson’s disease and aging links transcriptional dysregulation of energy metabolism to cell death. *Acta Neuropathol.* 122 75–86. 10.1007/s00401-011-0828-9 21541762

[B13] Garcia-Esparcia P. Hernández-Ortega K. Koneti A. Gil L. Delgado-Morales R. Castaño E. (2015). Altered machinery of protein synthesis is region- and stage-dependent and is associated with α-synuclein oligomers in Parkinson’s disease. *Acta Neuropathol. Commun.* 3:76. 10.1186/s40478-015-0257-4 26621506 PMC4666041

[B14] Gönczy P. Echeverri C. Oegema K. Coulson A. Jones S. J. M. Copley R. R. (2000). Functional genomic analysis of cell division in *C. elegans* using RNAi of genes on chromosome III. *Nature* 408 331–336. 10.1038/35042526 11099034

[B15] Harrington A. J. Hamamichi S. Caldwell G. A. Caldwell K. A. (2010). *C. elegans* as a model organism to investigate molecular pathways involved with Parkinson’s disease. *Dev. Dyn.* 239 1282–1295. 10.1002/dvdy.22231 20108318

[B16] Hartmann A. (2004). Postmortem studies in Parkinson’s disease. *Dial. Clin. Neurosci.* 6 281–293. 22033507 10.31887/DCNS.2004.6.3/ahartmann PMC3181805

[B17] Hashimoto K. Ishima T. (2011). Neurite outgrowth mediated by translation elongation factor eEF1A1: a target for antiplatelet agent cilostazol. *PLoS One* 6:e17431. 10.1371/journal.pone.0017431 21390260 PMC3046984

[B18] Hauser R. A. Slawek J. Barone P. Dohin E. Surmann E. Asgharnejad M. (2016). Evaluation of rotigotine transdermal patch for the treatment of apathy and motor symptoms in Parkinson’s disease. *BMC Neurol.* 16:90. 10.1186/s12883-016-0610-7 27267880 PMC4895976

[B19] Hawkins P. T. Anderson K. E. Davidson K. Stephens L. R. (2006). Signaling through Class I PI3Ks in mammalian cells. *Biochem. Soc. Trans.* 34 647–662. 10.1042/bst0340647 17052169

[B20] Kahns S. Lund A. Kristensen P. Knudsen C. R. Clark B. F. Cavallius J. (1998). The elongation factor 1 A-2 isoform from rabbit: cloning of the cDNA and characterization of the protein. *Nucleic Acids Res.* 26 1884–1890. 10.1093/nar/26.8.1884 9518480 PMC147499

[B21] Khwanraj K. Madlah S. Grataitong K. Dharmasaroja P. (2016). Comparative mRNA expression of eEF1A isoforms and a PI3K/Akt/mTOR pathway in a cellular model of parkinson’s disease. *Parkinsons Dis.* 2016:8716016. 10.1155/2016/8716016 26981313 PMC4769776

[B22] Lant B. Storey K. B. (2010). An overview of stress response and hypometabolic strategies in *Caenorhabditis elegans*: conserved and contrasting signals with the mammalian system. *Int. J. Biol. Sci.* 6 9–50. 10.7150/ijbs.6.9 20087441 PMC2808051

[B23] Latchoumycandane C. Anantharam V. Jin H. Kanthasamy A. Kanthasamy A. (2011). Dopaminergic neurotoxicant 6-OHDA induces oxidative damage through proteolytic activation of PKCδ in cell culture and animal models of Parkinson’s disease. *Toxicol. Appl. Pharmacol.* 256 314–323. 10.1016/j.taap.2011.07.021 21846476 PMC3205342

[B24] Li Z. Qi C.-F. Shin D.-M. Zingone A. Newbery H. J. Kovalchuk A. L. (2010). Eef1a2 promotes cell growth, inhibits apoptosis and activates JAK/STAT and AKT signaling in mouse plasmacytomas. *PLoS One* 5:e10755. 10.1371/journal.pone.0010755 20505761 PMC2873962

[B25] Licker V. Turck N. Kövari E. Burkhardt K. Côte M. Surini-Demiri M. (2014). Proteomic analysis of human substantia nigra identifies novel candidates involved in Parkinson’s disease pathogenesis. *Proteomics* 14 784–794. 10.1002/pmic.201300342 24449343

[B26] Lord C. E. N. Gunawardena A. H. L. A. N. (2012). Programmed cell death in *C. elegans*, mammals and plants. *Eur. J. Cell Biol.* 91 603–613. 10.1016/j.ejcb.2012.02.002 22512890

[B27] Maeda I. Kohara Y. Yamamoto M. Sugimoto A. (2001). Large-scale analysis of gene function in *Caenorhabditis elegans* by high-throughput RNAi. *Curr. Biol.* 11 171–176. 10.1016/S0960-9822(01)00052-5 11231151

[B28] Maulik M. Mitra S. Bult-Ito A. Taylor B. E. Vayndorf E. M. (2017). Behavioral phenotyping and pathological indicators of Parkinson’s disease in *C. elegans* models. *Front. Genet.* 8:77 10.3389/fgene.2017.00077 PMC5468440 28659967

[B29] Meulener M. Whitworth A. J. Armstrong-Gold C. E. Rizzu P. Heutink P. Wes P. D. (2005). *Drosophila* DJ-1 mutants are selectively sensitive to environmental toxins associated with Parkinson’s disease. *Curr. Biol.* 15 1572–1577. 10.1016/j.cub.2005.07.064 16139213

[B30] Moreno J. A. Radford H. Peretti D. Steinert J. R. Verity N. Martin M. G. (2012). Sustained translational repression by eIF2α-P mediates prion neurodegeneration. *Nature* 485 507–511. 10.1038/nature11058 22622579 PMC3378208

[B31] Nass R. Hall D. H. Miller D. M. III Blakely R. D. (2002). Neurotoxin-induced degeneration of dopamine neurons in *Caenorhabditis elegans*. *Proc. Natl. Acad. Sci. U.S.A.* 99 3264–3269. 10.1073/pnas.042497999 11867711 PMC122507

[B32] Offenburger S.-L. Ho X. Y. Tachie-Menson T. Coakley S. Hilliard M. A. Gartner A. (2018). 6-OHDA-induced dopaminergic neurodegeneration in *Caenorhabditis elegans* is promoted by the engulfment pathway and inhibited by the transthyretin-related protein TTR-33. *PLoS Genet.* 14:e1007125. 10.1371/journal.pgen.1007125 29346382 PMC5773127

[B33] Oster S. Radad K. Scheller D. Hesse M. Balanzew W. Reichmann H. (2014). Rotigotine protects against glutamate toxicity in primary dopaminergic cell culture. *Eur. J. Pharmacol.* 724 31–42. 10.1016/j.ejphar.2013.12.014 24365490

[B34] Pandey S. Srivanitchapoom P. (2017). Levodopa-induced dyskinesia: clinical features, pathophysiology, and medical management. *Ann. Indian Acad. Neurol.* 20 190–198. 10.4103/aian.AIAN_239_17 28904447 PMC5586110

[B35] Ray A. Zhang S. Rentas C. Caldwell K. A. Caldwell G. A. (2014). RTCB-1 mediates neuroprotection via XBP-1 mRNA splicing in the unfolded protein response pathway. *J. Neurosci.* 34 16076–16085. 10.1523/JNEUROSCI.1945-14.2014 25429148 PMC4244473

[B36] Sanyal S. Wintle R. F. Kindt K. S. Nuttley W. M. Arvan R. Fitzmaurice P. (2004). Dopamine modulates the plasticity of mechanosensory responses in *Caenorhabditis elegans*. *EMBO J.* 23 473–482. 10.1038/sj.emboj.7600057 14739932 PMC1271763

[B37] Sawin E. R. Ranganathan R. Horvitz H. R. (2000). *C. elegans* locomotory rate is modulated by the environment through a dopaminergic pathway and by experience through a serotonergic pathway. *Neuron* 26 619–631. 10.1016/S0896-6273(00)81199-X 10896158

[B38] Sun-Jung C. Lee H. Dutta S. Seog D. H. Moon I. S. (2012). Translation elongation factor-1A1 (eEF1A1) localizes to the spine by domain III. *BMB Rep.* 45 227–232. 10.5483/bmbrep.2012.45.4.227 22531132

[B39] Taymans J.-M. Nkiliza A. Chartier-Harlin M.-C. (2015). Deregulation of protein translation control, a potential game-changing hypothesis for Parkinson’s disease pathogenesis. *Trends Mol. Med.* 21 466–472. 10.1016/j.molmed.2015.05.004 26091824

[B40] Vera M. Pani B. Griffiths L. A. Muchardt C. Abbott C. M. Singer R. H. (2014). The translation elongation factor eEF1A1 couples transcription to translation during heat shock response. *eLife* 3:e03164. 10.7554/eLife.03164 25233275 PMC4164936

[B41] Zeng X.-S. Geng W.-S. Jia J.-J. (2018). Neurotoxin-induced animal models of parkinson disease: pathogenic mechanism and assessment. *ASN Neuro* 10:1759091418777438. 10.1177/1759091418777438 29809058 PMC5977437

[B42] Zhang J. Cai Q. Jiang M. Liu Y. Gu H. Guo J. (2017). Mesencephalic astrocyte-derived neurotrophic factor alleviated 6-OHDA-induced cell damage via ROS-AMPK/mTOR mediated autophagic inhibition. *Exp. Gerontol.* 89 45–56. 10.1016/j.exger.2017.01.010 28099881

[B43] Zhou Z. D. Selvaratnam T. Lee J. C. T. Chao Y. X. Tan E.-K. (2019). Molecular targets for modulating the protein translation vital to proteostasis and neuron degeneration in Parkinson’s disease. *Transl. Neurodegener.* 8:6. 10.1186/s40035-019-0145-0 30740222 PMC6360798

[B44] Zhu J. Hayakawa A. Kakegawa T. Kaspar R. L. (2001). Binding of the La autoantigen to the 5’ untranslated region of a chimeric human translation elongation factor 1A reporter mRNA inhibits translation in vitro. *Biochim. Biophys. Acta* 1521 19–29. 10.1016/S0167-4781(01)00277-9 11690632

