# Supplementary material for: Downregulation of eEF1A/EFT3-4 Enhances Dopaminergic Neurodegeneration After 6-OHDA Exposure in C. elegans Model
Source: Front Neurosci. 2020 Apr 16;14:303. doi: 10.3389/fnins.2020.00303 (PMC7212436; doi:10.3389/fnins.2020.00303)
Supplement: Supplementary file 1 [file Data_Sheet_1.DOCX]

**
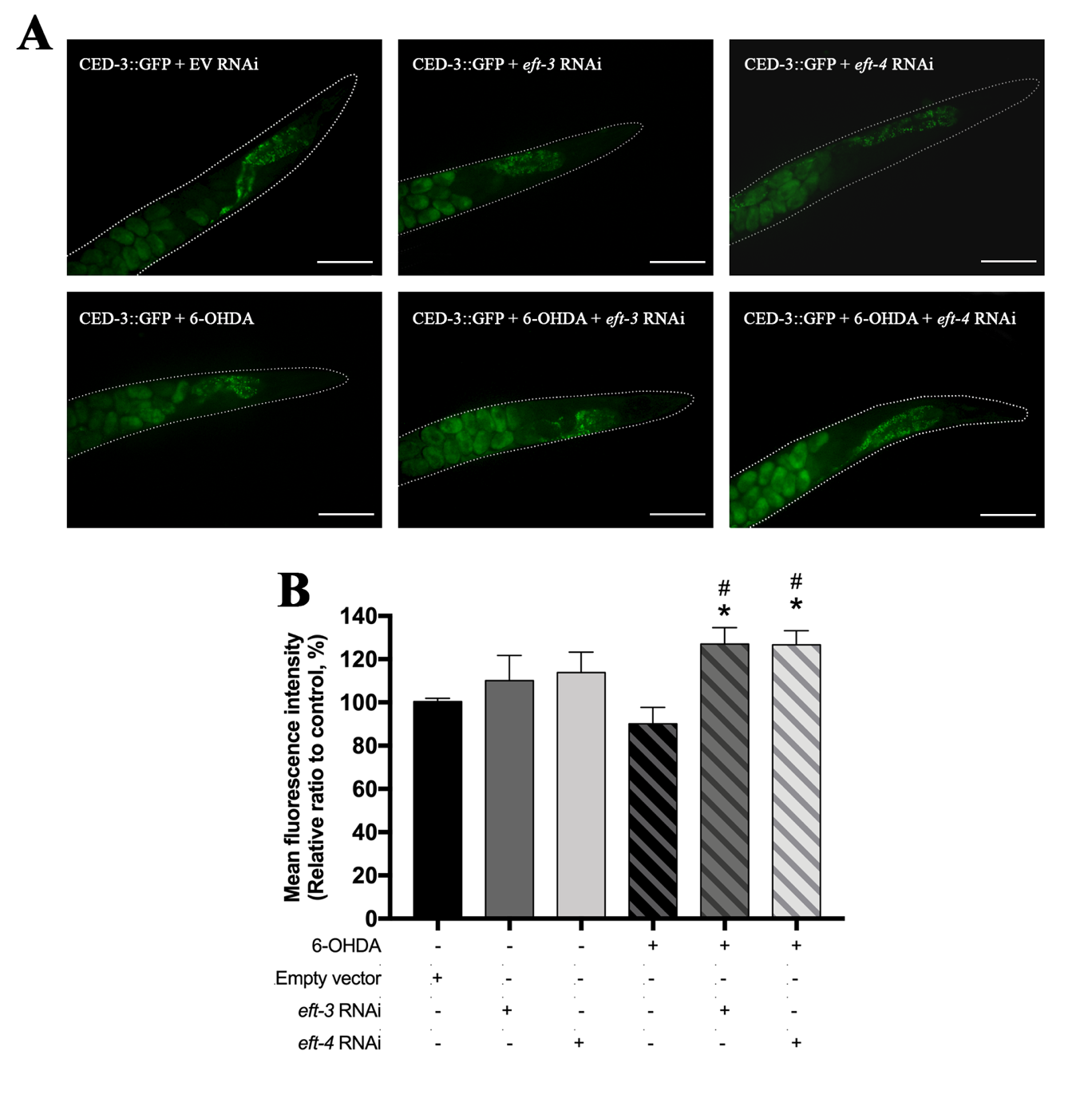
**

**Figure S1.** Effects of *eft-3*/*eft-4* RNAi and/or 6-OHDA exposure on CED-3 expression in CU394 strain. (A) GFP expression pattern of CED-3 in normal CU394 strain, *eft-3*/*eft-4* RNAi-treated, 6-OHDA-treated, and co-treatment between *eft-3/eft-4* RNAi and 6-OHDA-treated worms. Scale bar, 100 μm. (B) Graphical representation for MFI of GFP expression of CED-3 as measured by using ImageJ software. The asterisk (*) indicates a significant difference between the EV RNAi-treated and *eft-3/eft-4* RNAi and/or 6-OHDA-treated worms (*p*<0.05). The hash (#) indicates the significant difference between only 6-OHDA-treated and combined 6-OHDA with *eft-3* or *eft-4* RNAi-treated worms (*p*<0.05).
